# Improving meningitis surveillance and diagnosis with machine learning: Insights from São Paulo

**DOI:** 10.1371/journal.pdig.0000925

**Published:** 2025-07-10

**Authors:** Audêncio Victor, Diego Augusto Medeiros Santos, Eduardo Koerich Nery, Danilo Pereira Mori, Pamella Cristina de Carvalho Lucas, Denise Cammarota, Guillermo Leonardo Florez Montero, Fabiano Novaes Barcellos Filho, Ana Lúcia Frugis Yu, Telma Regina Marques Pinto Carvalhanas

**Affiliations:** 1 São Paulo State Health Department, Disease Control Coordination, Epidemiological Surveillance Center “Prof. Alexandre Vranjac,” Respiratory Disease Division, São Paulo, Brazil; 2 Public Health Postgraduate Program, School of Public Health, University of São Paulo, São Paulo, Brazil; 3 Department of Infectious Disease Epidemiology and International Health, London School of Hygiene & Tropical Medicine, London, United Kingdom; National Yang Ming Chiao Tung University, TAIWAN

## Abstract

**Introduction:**

Meningitis, an inflammatory condition of the membranes surrounding the brain and spinal cord, can be caused by various agents. Bacterial meningitis is particularly severe due to its high morbidity and mortality rates. This study aims to develop machine learning (ML) models to classify the aetiology of bacterial meningitis using data from the Notifiable Diseases Information System (SINAN) in São Paulo State, Brazil.

**Methods:**

Data were collected from the SINAN database, including sociodemographic variables, clinical symptoms, and cerebrospinal fluid (CSF) analyses. Five ML models Random Forest, LightGBM, XGBoost, CatBoost, and AdaBoost were applied to classify meningitis cases into bacterial, fungal, viral, and other types. Models were evaluated using metrics such as AUC-ROC, accuracy, precision, recall, F1-score, and MCC.

**Results:**

The CatBoost model demonstrated superior performance, achieving an AUC-ROC of 0.95 for binary classification (bacterial vs. non-bacterial) and 0.85 for multiclass classification (Neisseria meningitidis, Streptococcus pneumoniae, and Haemophilus influenzae). XGBoost and LightGBM also showed promising results with AUC-ROC scores of 0.94 and 0.92, respectively, for binary classification. The CatBoost model exhibited high sensitivity and reasonable specificity, highlighting its applicability in the rapid and accurate diagnosis of meningitis. SHAP analysis identified variables such as leukocyte count and the presence of petechiae as influential predictors in the models.

**Conclusion:**

ML algorithms, particularly CatBoost, XGBoost, and LightGBM, proved highly effective in the differential diagnosis of meningitis, offering a valuable tool for the rapid identification of meningitis types and bacterial serogroups. These techniques can be integrated into public health protocols to improve meningitis outbreak responses and optimize patient treatment.

## Author summary

Meningitis is a life-threatening disease that causes inflammation of the membranes surrounding the brain and spinal cord. It can be triggered by viruses, bacteria, fungi, or other pathogens, but bacterial meningitis is the most severe and requires urgent treatment. Rapidly distinguishing between bacterial and non-bacterial cases is crucial for both patient care and public health; however, even with laboratory tests, this differentiation can be complex and time-consuming. This challenge limits the effectiveness of clinical decisions and delays surveillance actions, especially in resource-constrained settings. In this study, we used machine learning (ML) models, advanced tools capable of identifying hidden patterns in large datasets, to improve the classification of meningitis cases. We analysed records from São Paulo, Brazil, encompassing demographic, clinical, and laboratory data. The CatBoost algorithm performed best, accurately identifying bacterial cases and the likely bacterial subtype involved. Our findings suggest that machine learning (ML) models can facilitate the early classification of meningitis cases, even when full laboratory confirmation is not yet available. These tools have the potential to enhance not only bedside decision-making but also the speed and precision of epidemiological surveillance, helping health systems respond more effectively to outbreaks and prioritize critical interventions.

## Introduction

Meningitis is an inflammation of the meninges, the membranes that cover the brain and spinal cord, triggered by bacterial, viral, fungal, parasitic, autoimmune, or neoplastic agents [1]. Among these aetiologies, bacterial meningitis is notably severe, posing a significant public health burden due to its high morbidity and mortality rates, especially in low and middle-income regions where access to healthcare resources is limited [2,3]. In 2019, approximately 236,000 meningitis-related deaths were reported globally, emphasising the need for precise and efficient diagnostic tools to manage this critical condition [3].

In Brazil, the incidence of meningitis is higher in children than in adults. Between 2010 and 2020, 182,126 meningitis cases were reported, 16,866 of which (9.26%) resulted in death, reflecting a case fatality rate of 9.26%. The fatality rate for bacterial meningitis can range from 3% to 19%, depending on the etiological agent and affected age group [4,5]. However, certain bacterial pathogens can be prevented through vaccination [6,7]. For instance, the pentavalent vaccine prevents infections caused by Haemophilus influenzae type B. It is administered at 2, 4, and 6 months of age, while the conjugated meningococcal vaccine, given at 3, 5, and 12 months, protects against infections by Neisseria meningitidis serogroup C [6–8].

Meningitis remains a public health issue with varying incidence across regions, especially in densely populated areas, such as São Paulo state in Brazil. Rapid differentiation between bacterial and viral aetiologies is crucial to reduce morbidity, optimize treatment, and prevent unnecessary use of antimicrobials, which contributes to bacterial resistance [9,10]. Current diagnostic methods, including cerebrospinal fluid (CSF) analysis by microscopy, culture, and PCR, are limited in availability in some contexts, hindering rapid clinical responses and epidemiological surveillance [11,12]. Furthermore, indiscriminate prescription of antibiotics for non-bacterial cases exacerbates antimicrobial resistance and complicates accurate diagnosis [10].

Given the complexity and limitations of traditional diagnostic methods, the rise of artificial intelligence (AI) models offers a promising approach to improve differential diagnosis of meningitis. AI models can process large volumes of clinical and laboratory data for rapid and accurate decision-making [13–15]. Machine learning (ML) models have shown high efficacy in distinguishing between bacterial and viral meningitis, optimizing epidemiological surveillance and case resolution [16]. However, there is a lack of studies exploring this approach in the Brazilian context, particularly using surveillance data to manage cases and prevent outbreaks. This study aims to develop an ML model to classify the etiology of bacterial meningitis using SINAN data from São Paulo State, aiming to enhance epidemiological surveillance and the effectiveness of control interventions.

## Methods

### Database and information sources

This is a retrospective study utilizing data from SINAN, encompassing all meningitis cases reported between 2007 and September 2024. SINAN collects detailed information on notifiable diseases, including meningitis, and is widely recognized for its importance in epidemiological surveillance and outbreak monitoring [17]. Previous studies, such as the analysis of bacterial meningitis in Brazil from 2009 to 2018, have highlighted SINAN’s contribution to understanding incidence and mortality patterns, validating its use for epidemiological studies [18].

### Ethics statement

This study was submitted to the Ethics Committee of the São Paulo State Health Department and approved under reference number CAAE 85820424.4.0000.0063 and opinion number 7.347.848.

### Outcome definition

Meningitis is characterized by a cerebrospinal fluid (CSF) leukocyte count exceeding five cells/mm³, accompanied by symptoms such as headache, nausea or vomiting, photophobia, neck stiffness, and fever above 38°C [19]. The primary outcome of this study is the etiology of bacterial meningitis, categorized into three main groups based on clinical and laboratory criteria: *Neisseria meningitidis* (meningococcus), *Haemophilus influenzae* (haemophilus), and *Streptococcus pneumoniae* (pneumococcus).

### Variables

Variables included in the model were selected based on their clinical and epidemiological relevance to the diagnosis and characterization of bacterial meningitis. Demographic variables included age (in years), race/ethnicity (White, yellow, Mixed, Black, and Indigenous), education level (Primary, secondary and higher), sex (male or female), residence region (urban, peri-urban, or rural). Patient comorbidities, such as HIV/AIDS, tuberculosis, immunosuppression (classified as “yes” or “no”), and contact history, were also included. Clinical symptoms such as headache, vomiting, neck stiffness, fever (>38°C), petechiae, meningeal signs, fontanelle bulging, seizures and coma, were classified as “present” or “absent”. Laboratory tests characterizing immune responses and CSF properties included red blood cell count, neutrophils, lymphocytes, eosinophils, CSF appearance, and protein and glucose concentrations.

### Machine learning model design

**Fig 1:**
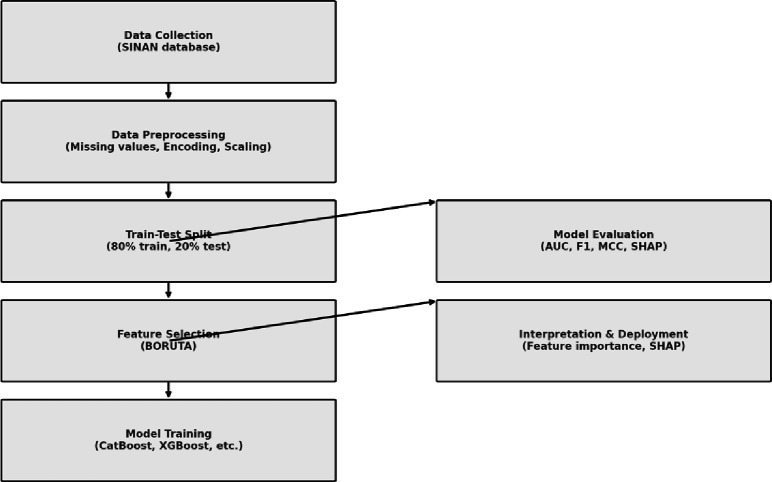
Simplified flowchart of the ML pipeline for classifying meningitis etiology using SINAN data.

The study aimed to enhance the diagnosis of bacterial meningitis using predictive models developed in two distinct stages. In the first stage, a binary classification model was built to distinguish between bacterial and non-bacterial meningitis. In the second stage, a multiclass classification model categorized bacterial meningitis cases into specific serogroups. A diverse set of ML algorithms, including ensemble methods, was employed to explore different predictive approaches and compare their performance. The methodology adhered to best practices for clinical data classification tasks, ensuring precision and robustness in the results [20,21], as illustrated in Fig 1 of the ML pipeline.

### Data preprocessing

Data preprocessing involved standardizing continuous variables using z-scores and encoding categorical variables using one-hot encoding. Predictors with over 20% missing values were removed, while those with less than 20% missing values were imputed using the mean, as recommended in health studies [22,23]. The dataset was split using a hold-out strategy, with 80% allocated for training and 20% for testing.

### Algorithm selection

Four machine learning algorithms were tested: CatBoost [24], XGBoost [25], LightGBM [26] and Random Forest [27]. These algorithms were chosen for their robustness in classification tasks. CatBoost, XGBoost, and LightGBM were implemented using their respective Python libraries, with the overall development conducted using Python, employing libraries such as scikit-learn for model building, pandas for data manipulation, and matplotlib for visualization [28].

### Hyperparameter selection

Hyperparameter optimization was performed on the training set using 5-fold stratified cross-validation and GridSearchCV [29]. Due to significant class imbalance, where the minority class represented less than 25% of total outcomes, the Synthetic Minority Over-sampling Technique (SMOTE) was applied. Additionally, the BORUTA feature selection method was employed in the training set [30].

### Model evaluation

The models were evaluated on the test set using metrics such as the area under the receiver operating characteristic curve (AUC-ROC), sensitivity, specificity, precision, F1-score, and Matthew’s correlation coefficient (MCC). SHAP values were calculated to interpret and evaluate the contribution of each predictor to the outcome in the test set [31–33] adhering to the Transparent Reporting of a Multivariable Prediction Model for Individual Prognosis or Diagnosis (TRIPOD) guidelines [34].

### Statistical analysis

Descriptive statistics were used to summarize the characteristics of the study population. Categorical variables were presented as absolute and relative frequencies, and continuous variables were expressed as medians and interquartile ranges (Q1–Q3) due to their non-normal distribution. Differences between two groups (e.g., bacterial vs. non-bacterial meningitis, Table 1) were assessed using the Wilcoxon rank sum test for continuous variables and the Chi-square test or Fisher’s exact test, as appropriate, for categorical variables. For comparisons involving three or more groups (e.g., different bacterial meningitis types: Haemophilus, Meningococcus, Other Bacteria, Table 2), the Kruskal–Wallis rank sum test was used for continuous variables, and the Chi-square test or Fisher’s exact test was used for categorical variables. A p-value < 0.05 was considered statistically significant.

**Table 1:** Demographic and clinical features of patients with bacterial and non-Bacterial Meningitis in São Paulo, Brazil (2007–2024).

Features	Overall N = 101,224^1^	Bacterial N = 27,476^1^	Nonbacterial N = 73,748^1^
**Age**	7 (2-29)	20 (3-46)	5 (2-21)
**Sex**			
*Feminine*	42,506 (42%)	11,708 (42.6%)	30,798 (41.8%)
*Masculine*	58,692 (58%)	15,757 (57.4%)	42,935 (58.2%)
**Raçe**			
*Yellow*	717 (0.7%)	156 (0.6%)	561 (0.8%)
*White*	55,918 (55.2%)	15,257 (55.5%)	40,661 (55.1%)
*Ignored*	24,552 (24.3%)	4,181 (15.2%)	20,371 (27.6%)
*Indíginous*	146 (0.1%)	34 (0.1%)	112 (0.2%)
*Mixed race*	16,405 (16.2%)	6,280 (22.9%)	10,125 (13.7%)
*Black*	3,486 (3.4%)	1,568 (5.7%)	1,918 (2.6%)
**Education Level**			
*Primary*	13,741 (13.6%)	5,140 (18.7%)	8,601 (11.7%)
*Secondary*	6,341 (6.3%)	2,590 (9.4%)	3,751 (5.1%)
*Higher*	2,461 (2.4%)	690 (2.5%)	1,771 (2.4%)
*Ignored*	29,157 (28.8%)	9,847 (35.8%)	19,310 (26.2%)
*Not Aplicable*	49,524 (48.9%)	9,209 (33.5%)	40,315 (54.7%)
**Region of residence**			
*Ignored*	3,452 (3.4%)	1,150 (4.2%)	2,302 (3.1%)
*Periurban*	268 (0.3%)	123 (0.4%)	145 (0.2%)
*Rural*	1,245 (1.2%)	504 (1.8%)	741 (1%)
*Urban*	96,259 (95.1%)	25,699 (93.5%)	70,560 (95.7%)
**AIDS**			
*Yes*	2,461 (3%)	871 (3.9%)	1,590 (2.7%)
*No*	79,719 (97%)	21,608 (96.1%)	58,111 (97.3%)
**Imunosuppresessed**			
*Yes*	1,156 (1.4%)	492 (2.2%)	664 (1.1%)
*No*	80,240 (98.6%)	21,850 (97.8%)	58,390 (98.9%)
**Tuberculosis**			
*Yes*	702 (0.9%)	491 (2.2%)	211 (0.4%)
*No*	81,447 (99.1%)	22,061 (97.8%)	59,386 (99.6%)
**Headache**			
*No*	28,044 (29.6%)	8,639 (34.8%)	19,405 (27.7%)
*Yes*	66,788 (70.4%)	16,218 (65.2%)	50,570 (72.3%)
**Fever**			
*No*	18,201 (18.4%)	4,295 (16.1%)	13,906 (19.3%)
*Yes*	80,622 (81.6%)	22,313 (83.9%)	58,309 (80.7%)
**Vomiting**			
*No*	38,770 (39.6%)	10,104 (38.7%)	28,666 (40%)
*Yes*	59,019 (60.4%)	15,991 (61.3%)	43,028 (60%)
**Seizures**			
*No*	86,304 (89.6%)	21,319 (83.2%)	64,985 (91.9%)
*Yes*	10,031 (10.4%)	4,312 (16.8%)	5,719 (8.1%)
**Neck stifness**			
*No*	63,799 (66.5%)	14,761 (58.3%)	49,038 (69.4%)
*Yes*	32,136 (33.5%)	10,551 (41.7%)	21,585 (30.6%)
**Meningeal signs**			
*No*	88,954 (94.7%)	22,335 (91.9%)	66,619 (95.7%)
*Yes*	4,965 (5.3%)	1,973 (8.1%)	2,992 (4.3%)
**Fontanelle bulging**			
*No*	93,334 (97.6%)	24,242 (95.9%)	69,092 (98.2%)
*Yes*	2,309 (2.4%)	1,048 (4.1%)	1,261 (1.8%)
**Coma**			
*No*	92,490 (96.6%)	22,905 (90.3%)	69,585 (98.9%)
*Yes*	3,230 (3.4%)	2,459 (9.7%)	771 (1.1%)
**Petechiae**			
*No*	87,335 (90.5%)	17,887 (68.7%)	69,448 (98.5%)
*Yes*	9,209 (9.5%)	8,134 (31.3%)	1,075 (1.5%)
**CSF appearance**			
*Hemorrhagic*	2,195 (2.2%)	666 (2.4%)	1,529 (2.1%)
*Ignored*	7,561 (7.5%)	3,785 (13.8%)	3,776 (5.1%)
*Clear*	58,299 (57.6%)	6,696 (24.4%)	51,603 (70%)
*Other*	2,948 (2.9%)	464 (1.7%)	2,484 (3.4%)
*Purulent*	1,439 (1.4%)	1,266 (4.6%)	173 (0.2%)
*Couldy*	25,563 (25.3%)	12,858 (46.8%)	12,705 (17.2%)
*Xanthochromic*	3,219 (3.2%)	1,741 (6.3%)	1,478 (2%)
**Cytological chemistry tests**			
*Red blood cells*	100 (0-170)	106 (11, 490)	5 (0-70)
*Neutrophils*	30 (8-70)	82 (60-92)	20 (6-46)
*Glucose*	54 (42-64)	24 (7-55)	56 (49-65)
*Leukocytes*	104 (30-358)	700 (80-3,250)	80 (27-210)
*Proteins*	49 (29-113)	197 (79-446)	40 (27-67)
*Monocytes*	6 (2-14)	3 (0-7)	8 (3-15)
*Lymphocytes*	60 (21, 83)	12 (5, 32)	70 (40-86)
*Chloride*	120 (113-127)	117 (110-124)	121 (114-128)

^1^Median (Q1, Q3); n (%). All variables were significantly associated with meningitis etiology (p < 0.05).

^2^Wilcoxon rank sum test; Pearson’s Chi-squared test.

**Table 2:** Demographic and clinical features by bacterial meningitis type (Haemophilus, Meningococcus, Other Bacteria, Pneumococcus) in São Paulo, Brazil (2007–2024).

Features	Overall N = 27,476^1^	Haemophilus N = 874^1^	Meningococcus N = 12,812^1^	Other Bacteria N = 6,021^1^	Pneumococcus N = 7,769^1^
**Age**	20 (3- 46)	3(0 - 34)	11(3- 29)	33(4 - 53)	37(9 - 55)
**Sex**					
*Feminine*	11,708 (42.6)	391 (44.8)	5,633 (44)	2,442 (40.6)	3,242 (41.8)
*Masculine*	15,757 (57.4)	482 (55.2)	7,179 (56)	3,573 (59.4)	4,523 (58.2)
**Raçe**					
*Yellow*	156 (0.6)	0 (0)	64 (0.5)	41 (0.7)	51 (0.7)
*White*	15,257 (55.5)	516 (59)	6,792 (53)	3,665 (60.9)	4,284 (55.1)
*Ignored*	4,181 (15.2)	123 (14.1)	2,012 (15.7)	844 (14)	1,202 (15.5)
*Indiginous*	34 (0.1)	1 (0.1)	15 (0.1)	6 (0.1)	12 (0.2)
*Mixed race*	6,280 (22.9)	190 (21.7)	3,210 (25.1)	1,120 (18.6)	1,760 (22.7)
*Black*	1,568 (5.7)	44 (5)	719 (5.6)	345 (5.7)	460 (5.9)
**Education Level**					
*Primary*	5,140 (18.7)	98 (11.2)	2,361 (18.4)	1,252 (20.8)	1,429 (18.4)
*Secondary*	2,590 (9.4)	56 (6.4)	1,145 (8.9)	556 (9.2)	833 (10.7)
*Higher*	690 (2.5)	22 (2.5)	280 (2.2)	179 (3)	209 (2.7)
*Ignored*	9,847 (35.8)	203 (23.2)	3,779 (29.5)	2,322 (38.6)	3,543 (45.6)
*Not Applicable*	9,209 (33.5)	495 (56.6)	5,247 (41)	1,712 (28.4)	1,755 (22.6)
**Residence region**					
*Ignored*	1,150 (4.2)	31 (3.5)	522 (4.1)	282 (4.7)	315 (4.1)
*Periurbana*	123 (0.4)	2 (0.2)	57 (0.4)	33 (0.5)	31 (0.4)
*Rural*	504 (1.8)	15 (1.7)	188 (1.5)	153 (2.5)	148 (1.9)
*Urban*	25,699 (93.5)	826 (94.5)	12,045 (94)	5,553 (92.2)	7,275 (93.6)
**AIDS**					
*Yes*	871 (3.9)	10 (1.3)	114 (1.1)	556 (11)	191 (3.1)
*No*	21,608 (96.1)	732 (98.7)	10,375 (98.9)	4,510 (89)	5,991 (96.9)
**Imunosuppresessed**					
*Yes*	4920 (2.2)	22.0 (3.0)	123.0 (1.2)	190.0 (3.8)	157.0 (2.6)
*No*	21,850.0 (97.8)	722.0 (97.0)	10,350(98.8)	4,780.0 (96.2)	5,998(97.4)
**Tuberculosis**					
*Yes*	491.0 (2.2)	1 (0.1)	28 (0.3)	439 (8.7)	23 (0.4)
*No*	22,061 (97.8)	747 (99.9)	10,559 (99.7)	4,587 (91.3)	6,168 (99.6)
**Headache**					
*No*	8,639 (34.8)	334 (43.5)	3,872 (33.4)	2,340 (43.2)	2,093 (29.6)
*Yes*	16,218 (65.2)	433 (56.5)	7,727 (66.6)	3,077 (56.8)	4,981 (70.4)
**Fever**					
*No*	4,295 (16.1)	142 (16.8)	1,177 (9.4)	1,454 (25.3)	1,522 (20.4)
*Yes*	22,313 (83.9)	705 (83.2)	11,374 (90.6)	4,292 (74.7)	5,942 (79.6)
**Vomiting**					
*No*	10,104 (38.7)	269 (32.2)	3,597 (29.2)	3,228 (57.5)	3,010 (41.1)
*Yes*	15,991 (61.3)	567 (67.8)	8,727 (70.8)	2,387 (42.5)	4,310 (58.9)
**Seizures**					
*No*	21,319 (83.2)	679 (82.8)	10,657 (89.1)	4,568 (81.9)	5,415 (74.5)
*Yes*	4,312 (16.8)	141 (17.2)	1,309 (10.9)	1,010 (18.1)	1,852 (25.5)
**Neck stiffness**					
*No*	14,761 (58.3)	468 (57.9)	6,331 (53.3)	3,994 (72.7)	3,968 (55.7)
*Yes*	10,551 (41.7)	340 (42.1)	5,549 (46.7)	1,502 (27.3)	3,160 (44.3)
**Meningeal signs**					
*No*	22,335 (91.9)	724 (93.5)	10,140 (89.6)	5,167 (96.3)	6,304 (92)
*Yes*	1,973 (8.1)	50 (6.5)	1,177 (10.4)	197 (3.7)	549 (8.0)
**Fontanelle bulging**					
*No*	24,242 (95.9)	742 (91.4)	11,339 (96)	5,343 (96.6)	6,818 (95.5)
*Yes*	1,048 (4.1)	70 (8.6)	470 (4)	190 (3.4)	318 (4.5)
**Coma**					
*No*	22,905 (90.3)	771 (95.4)	10,901 (92)	5,024 (90.6)	6,209 (86.6)
*Yes*	2,459 (9.7)	37 (4.6)	943 (8)	522 (9.4)	957 (13.4)
**Petechiae**					
*No*	17,887 (68.7)	755 (92.5)	4,927 (39.6)	5,318 (95.8)	6,887 (95.4)
*Yes*	8,134 (31.3)	61 (7.5)	7,505 (60.4)	235 (4.2)	333 (4.6)
**CSF appearance**					
*Hemorrhagic*	666 (2.4)	11 (1.3)	198 (1.5)	329 (5.5)	128 (1.6)
*Ignored*	3,785 (13.8)	107 (12.2)	2,482 (19.4)	444 (7.4)	752 (9.7)
*Clear*	6,696 (24.4)	190 (21.7)	3,279 (25.6)	1,922 (31.9)	1,305 (16.8)
*Other*	464 (1.7)	13 (1.5)	127 (1.0)	178 (3.0)	146 (1.9)
*Purulent*	1,266 (4.6)	32 (3.7)	694 (5.4)	124 (2.1)	416 (5.4)
*Couldy*	12,858 (46.8)	454 (51.9)	5,524 (43.1)	2,479 (41.2)	4,401 (56.6)
*Xanthochromic*	1,741 (6.3)	67 (7.7)	508 (4)	545 (9.1)	621 (8)
**Cytological chemistry tests**					
*Red blood cells*	106(11 - 490)	145(20 - 584)	118(10 - 480)	85(9 - 685)	110(17 -442)
*Neutrophils*	82(60 - 92)	81(64- 90)	86(67- 93)	69(28 - 86)	85(70 - 92)
*Glucose*	24(7 - 55)	20(6 - 44)	31(10 - 60)	36(14 -58)	10(5 - 37)
*Leukocytes*	700(80, 3-250)	1,365(321.5-4,135)	1,102(38-4,320)	265.50 (64-1,194)	930(160-3,360)
*Proteins*	197(79-446)	175(95-328)	162(45-372)	160(74-352)	311(154-622.5)
*Monocytes*	3(0-7)	3(1-8)	2(0-6)	4(1-9)	3(0-6)
*Lymphocytes*	12(5-32)	13(6-31)	10(4-24)	25(10-64)	11(5-25)
*Chloride*	117(110-124)	119(111.5-125.5)	118(111-125)	117(109-125)	116(110-123)

^1^Median (Q1, Q3); n (%), All variables were significantly associated with meningitis type (p < 0.05).

^2^Kruskal-Wallis rank sum test; Pearson’s Chi-squared test.

## Results

### Demographic and clinical characteristics

As presented in Table 1, the demographic and clinical characteristics of patients with meningitis revealed significant differences between those with bacterial and non-bacterial meningitis. The median age was significantly higher in bacterial meningitis cases (20 years) compared to non-bacterial meningitis cases (5 years). A male predominance was observed in both groups. White ethnicity was the most common across all groups, with similar proportions in bacterial and non-bacterial meningitis cases (P < 0.001). Most cases occurred in urban areas, and conditions such as AIDS and immunosuppression were more prevalent among patients with bacterial meningitis (P < 0.001).

Analysis of variations associated with specific bacterial etiological agents showed that the median age of patients varied significantly, being lowest for *Haemophilus influenzae* (3 years) and highest for *Streptococcus pneumoniae* (37 years) (P < 0.001), as detailed in Table 2. Male predominance was more pronounced in *Streptococcus pneumoniae* infections (58.2%) (P < 0.001). White ethnicity predominated, especially in cases caused by other bacteria (60.9%) (P < 0.001). Urban areas were the most common residence among patients, while conditions such as AIDS and immunosuppression were particularly prevalent in cases caused by other bacteria (P < 0.001).

### Overall model performance

**Fig 2:**
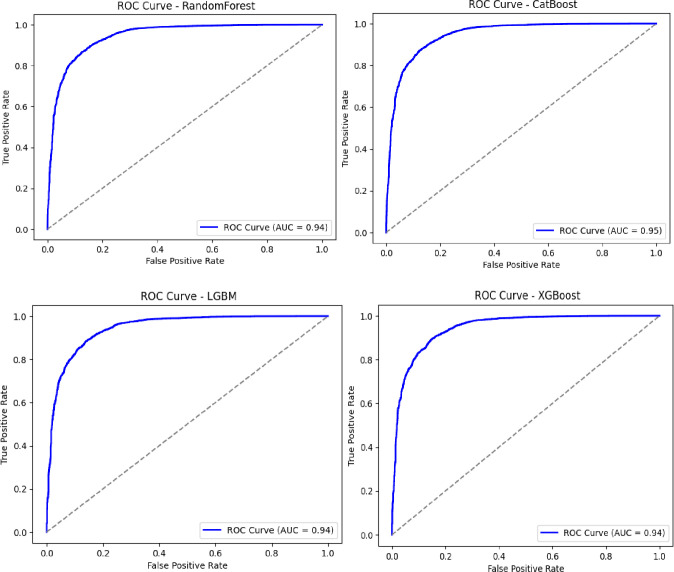
Predictive performance of algorithms for binary classification of bacterial vs. non-bacterial meningitis (AUC-ROC).

**Fig 3:**
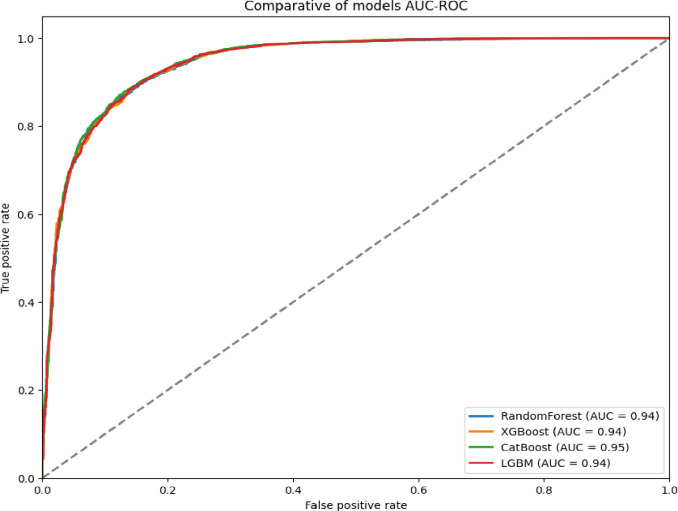
Predictive performance of algorithms for binary classification of bacterial vs. non-bacterial meningitis: weighted average (AUC-ROC).

**Fig 4:**
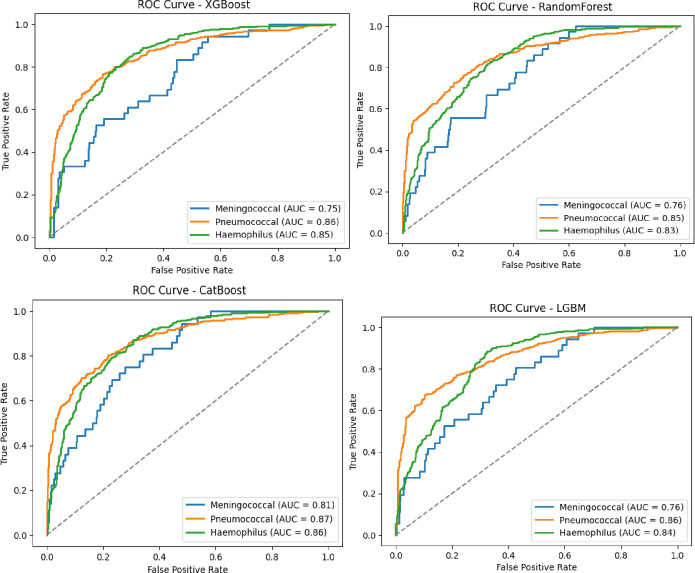
Predictive performance of algorithms for multiclass classification by serogroup of bacterial meningitis (AUC-ROC).

**Fig 5:**
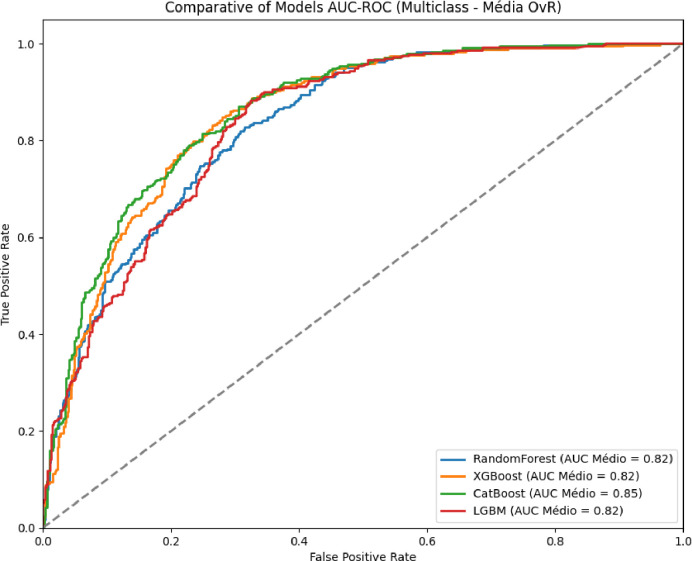
Predictive performance of algorithms for multiclass classification by serogroup of bacterial meningitis: weighted average per algorithm (AUC-ROC).

As illustrated in Figs 2 and 3, the ML models Random Forest, LightGBM, XGBoost, and CatBoost were evaluated for their ability to distinguish between bacterial and non-bacterial meningitis. The CatBoost model achieved the best overall performance, with an AUC-ROC of 0.95, followed by XGBoost, Random Forest, and LightGBM, which achieved AUC-ROC scores of 0.94, 0.92, and 0.91, respectively. Table 3, for multiclass classification, aimed at identifying the three main bacterial serogroups (Neisseria meningitidis, Streptococcus pneumoniae, and Haemophilus influenzae), CatBoost again outperformed other models with an AUC-ROC of 0.85. XGBoost and LightGBM followed, with AUC-ROC scores of 0.82 and 0.80, respectively (Figs 4 and 5).

**Table 3:** Performance metrics of best algorithms for multiclass classification by serogroup of bacterial meningitis with hyperparameter tuning.

Model	AUC-ROC	Accuracy	Recall	Specificity	Precision	F1	MCC
RandomForest	0,82	0,71	1,00	0,16	0,35	0,48	0,48
XGBoost	0,82	0,76	1,00	0,10	0,34	0,47	0,52
CatBoost	0,85	0,76	1,00	0,20	0,36	0,49	0,53
LGBM	0,82	0,69	1,00	0,14	0,35	0,48	0,47

Table 4 summarizes the performance metrics, highlighting the superiority of CatBoost in sensitivity (1.00), while specificity varied between 0.09 and 0.17. Although all models exhibited high precision and consistent F1-scores, CatBoost achieved a better balance among these metrics, as evidenced by its Matthews correlation coefficient (MCC) of 0.27.

**Table 4:** Performance metrics of best algorithms for binary classification of bacterial vs. non-bacterial meningitis with hyperparameter tuning.

Model	AUC-ROC	Accuracy	Recall	Specificity	Precision	F1	MCC
RandomForest	0,94	0,71	1,00	0,09	0,71	0,83	0,25
XGBoost	0,94	0,74	1,00	0,17	0,73	0,84	0,35
CatBoost	0,95	0,72	1,00	0,10	0,71	0,83	0,27
LGBM	0,94	0,72	1,00	0,11	0,71	0,83	0,28

### Variable importance analysis (SHAP)

**Fig 6:**
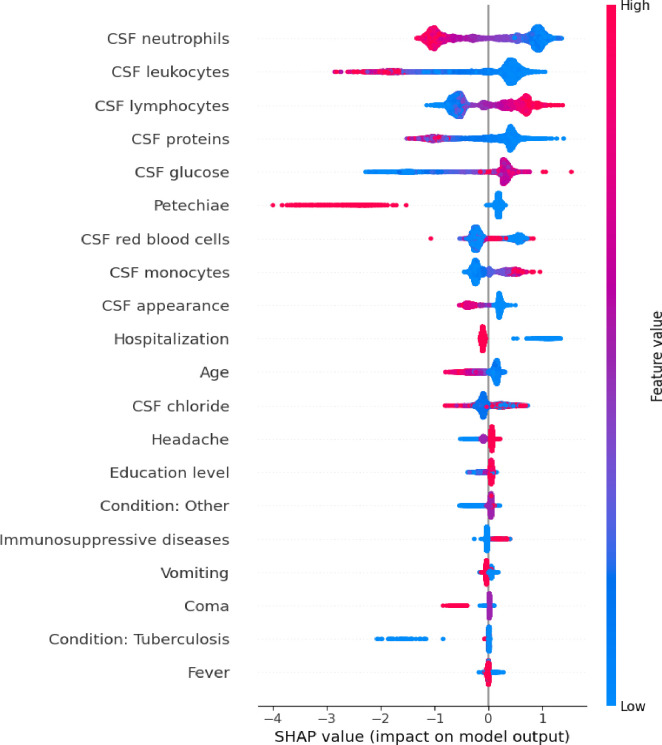
SHAP analysis showing feature contributions for diagnosing bacterial vs. non-bacterial meningitis using CatBoost.

SHAP analysis identified the most influential variables in the models’ performance. For binary classification, neutrophil, leukocyte, and lymphocyte counts in CSF emerged as the most critical predictors. Protein and glucose concentrations in CSF, along with the presence of petechiae, were also significant contributors. The contribution of these variables to CatBoost model performance is illustrated in Fig 6.

## Discussion

This study evaluated the efficacy of ML models in the differential diagnosis of meningitis, with a specific focus on ensemble models such as CatBoost, XGBoost, and LightGBM. The results indicate that these models can provide faster, and more accurate diagnoses compared to traditional methods. Specifically, the CatBoost model excelled in efficiently handling categorical variables and minimizing overfitting, demonstrating remarkable sensitivity and specificity. This study highlights the significant potential of integrating artificial intelligence into public health to enhance responses to infectious diseases.

The ability of these models to process large datasets and identify complex, non-linear patterns offer a valuable tool for healthcare professionals, particularly in regions where access to advanced diagnostics is limited. The implementation of these algorithms can facilitate triage and early diagnosis, enhancing the effectiveness of medical interventions and reducing the burden on healthcare systems [14,15,35,36].

In classification problems, particularly those involving imbalanced datasets such as this one, relying solely on traditional metrics (e.g., sensitivity or specificity) may not provide a complete picture of model performance. Therefore, this study also employed composite evaluation metrics, including the area under the receiver operating characteristic curve (AUROC) and the F1-score, which offer a more balanced assessment of classifier performance. AUROC reflects the model’s overall ability to distinguish between classes at various thresholds. At the same time, F1-score harmonizes precision and recall, which is particularly valuable when the cost of false positives and false negatives must be jointly considered. These metrics are fundamental in the health domain, where decisions must weigh both timely detection (sensitivity) and diagnostic accuracy (specificity). While the CatBoost model achieved a sensitivity of 1.0critical for not missing true bacterial meningitis cases, the AUROC and F1-score provided additional insight into its performance trade-offs. Strategies to improve specificity, such as hybrid models that incorporate clinical criteria alongside ML outputs, should be considered for practical implementation.

These findings align with a recent systematic review by Ghaddaripouri et al. (2024), which assessed 16 studies using ML for meningitis prediction and diagnosis. The review found that ML algorithms, especially ensemble and tree-based models, outperformed traditional methods in diagnostic accuracy and risk stratification. The authors noted the increasing importance of ML tools, emphasising their potential for scalable public health decision support. Our study adds to this field by applying advanced ensemble algorithms and providing a framework for their integration into surveillance systems. Additionally, while most studies in the review focused on binary classifications (bacterial vs. viral), our two-stage ML approach differentiates between major bacterial etiologies, marking a significant advancement. [13,29,37]. Ensemble models such as CatBoost have demonstrated superior performance across various medical applications, from cancer diagnosis to infectious disease outbreak predictions [24,38]. Moreover, ensemble algorithms, particularly boosting techniques, are increasingly recognized as state-of-the-art for tabular data, which predominates in the healthcare sector [20,21,39]. The inclusion of these models in epidemiological studies confirms the trend that ML techniques not only complement but often surpass traditional data analysis methods in healthcare.

Despite these promising results, this study has several limitations. Our dataset drives from from a single source, SINAN, which may not be representative of other regions or populations. Additionally, the quality and consistency of data collected through surveillance systems can vary, potentially affecting ML model performance [40]. The generalizability of these findings is another concern, as the models were trained and tested on a specific dataset. Future studies should evaluate the robustness of these models across diverse geographic and demographic contexts to ensure universal applicability [36,41].

Another limitation is the trade-off between sensitivity and specificity in the binary classification model. While high sensitivity is crucial in meningitis surveillance to avoid missing critical cases, the low specificity results in a higher rate of false positives, leading to unnecessary treatments or follow-ups. The class imbalance in the dataset, with fewer non-bacterial cases, likely contributed to this issue despite applying techniques to mitigate it SMOTE. Further research should explore post-processing strategies or hybrid clinical-ML decision tools that can improve specificity while maintaining acceptable sensitivity. Furthermore, interpreting variable importance using SHAP analysis should be approached with caution. The importance attributed to certain features does not necessarily imply causality, which may lead to misleading conclusions if not supported by clinical knowledge and additional studies [42]. To overcome these limitations and expand knowledge in this field, multicentre studies that include data from various regions and populations are recommended. Such studies would not only improve the generalization of ML models but also provide a more comprehensive understanding of meningitis dynamics in different contexts. Additionally, integrating imaging and genomic data could enrich these models, enabling a more holistic and detailed approach to diagnosing meningitis [43,44].

The adoption of ML models in clinical practice also requires ethical considerations, particularly regarding data privacy and informed consent. As these technologies advance, it is crucial to develop clear guidelines for their implementation, ensuring they are used ethically and responsibly [45,46]

## Conclusion

ML algorithms, particularly CatBoost, XGBoost, and LightGBM, proved to be highly effective in the differential diagnosis of meningitis using SINAN data from São Paulo. These models demonstrated high accuracy and sensitivity, offering valuable tools for the rapid identification of meningitis types and bacterial serogroups. Such techniques can be integrated into public health protocols to enhance outbreak response and optimize patient treatment. Despite the promising results, it is essential to validate these models across different populations to ensure their broader applicability. Future studies should validate these models in other regions and populations. Integrating clinical, genomic, or imaging data may also enhance predictive accuracy.

## Supporting information

S1 Fig Confusion matrix for binary classification of bacterial vs. non-bacterial meningitis on test data. (DOCX)

S2 Fig Confusion matrix for multiclass classification of bacterial meningitis by serogroup on test data. (DOCX)

S1 Table Total sample and train/test split by bacterial meningitis subtype (N = 27,476). (DOCX)

S2 Table Feature groups and examples of variables used in model training. (DOCX)
