# Supplementary material for: Improving meningitis surveillance and diagnosis with machine learning: Insights from São Paulo
Source: PLOS Digit Health. 2025 Jul 10;4(7):e0000925. doi: 10.1371/journal.pdig.0000925 (PMC12244477; doi:10.1371/journal.pdig.0000925)

**S1 Fig: Confusion matrix for binary classification of bacterial vs. non-bacterial meningitis on test data.**


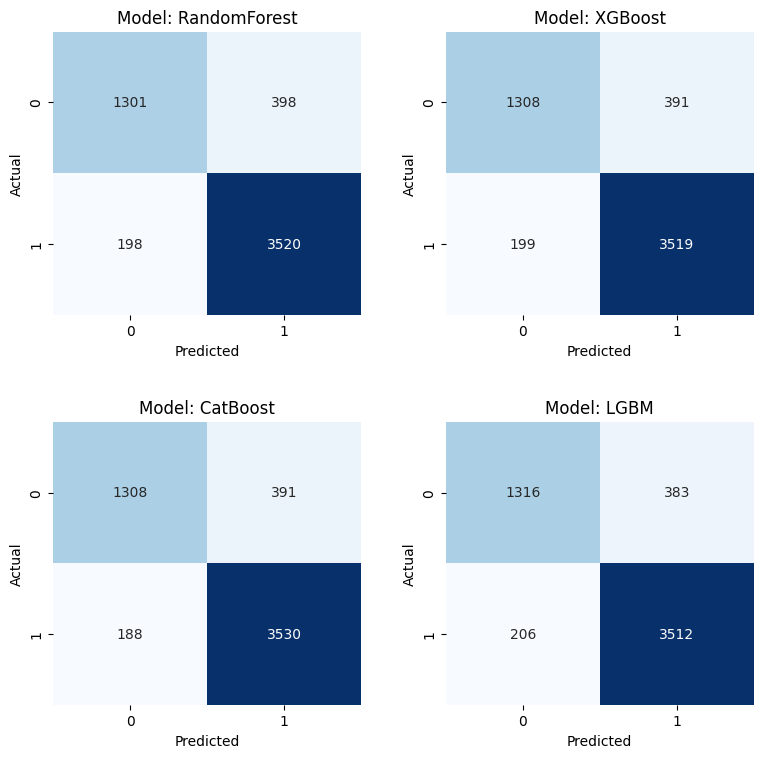

Supplement: S1 Fig — (DOCX) [file pdig.0000925.s001.docx]
