# Supplementary material for: Improving meningitis surveillance and diagnosis with machine learning: Insights from São Paulo
Source: PLOS Digit Health. 2025 Jul 10;4(7):e0000925. doi: 10.1371/journal.pdig.0000925 (PMC12244477; doi:10.1371/journal.pdig.0000925)
Supplement: S1 Table — (DOCX) [file pdig.0000925.s003.docx]

**S1 Table. Total sample and train/test split by bacterial meningitis subtype (N = 27,476)**

| **Bacterial Meningitis Subtype** | **Total (N)** | **Training Set (80%)** | **Test Set (20%)** |
| --- | --- | --- | --- |
| Haemophilus | 526 | 421 | 105 |
| Meningococcus | 12,321 | 9,857 | 2,464 |
| Pneumococcus | 7,790 | 6,232 | 1,558 |
| Other Bacteria | 6,839 | 5,471 | 1,368 |
