# Supplementary material for: Improving meningitis surveillance and diagnosis with machine learning: Insights from São Paulo
Source: PLOS Digit Health. 2025 Jul 10;4(7):e0000925. doi: 10.1371/journal.pdig.0000925 (PMC12244477; doi:10.1371/journal.pdig.0000925)
Supplement: S2 Table — (DOCX) [file pdig.0000925.s004.docx]

**S2 Table. Feature groups and examples of variables used in model training**

| **Feature Group** | **Examples of Variables** |
| --- | --- |
| Sociodemographic | Age (continuous), Sex (female/male), Race/Ethnicity (White, Black, Mixed, Indigenous, Asian), Education level, Region (Urban/Rural) |
| Clinical Presentation | Fever, Headache, Vomiting, Seizures, Neck stiffness, Meningeal signs, Bulging fontanelle, Coma, Petechiae |
| Comorbidities | History of Tuberculosis, AIDS, HIV infection |
| CSF Macroscopic | CSF appearance (Clear, Turbid, Hemorrhagic, Purulent and Xanthochromic ) |
| CSF Cytology & Biochemistry | Red and white blood cell count, Neutrophils, Lymphocytes, Glucose, Protein, Chloride levels |
